# Comparison Between Non–vitamin K Antagonist Oral Anticoagulants and Low-Molecular-Weight Heparin in Asian Individuals With Cancer-Associated Venous Thromboembolism

**DOI:** 10.1001/jamanetworkopen.2020.36304

**Published:** 2021-02-03

**Authors:** Dong-Yi Chen, Chi-Nan Tseng, Ming-Jer Hsieh, Wen-Ching Lan, Cheng-Keng Chuang, See-Tong Pang, Shao-Wei Chen, Tien-Hsing Chen, Shang-Hung Chang, I-Chang Hsieh, Pao-Hsien Chu, Ming-Shien Wen, Jen-Shi Chen, John Wen-Cheng Chang, Lai-Chu See, Wen-Kuan Huang

**Affiliations:** 1 Division of Cardiology, Department of Internal Medicine, Chang Gung Memorial Hospital at Linkou, Chang Gung University College of Medicine, Taoyuan, Taiwan; 2 Cardio-Oncology Program, Chang Gung Memorial Hospital at Linkou, Taoyuan, Taiwan; 3 Department of Thoracic and Cardiovascular Surgery, Chang Gung Memorial Hospital at Linkou, Chang Gung University College of Medicine, Taoyuan, Taiwan; 4 Center for Big Data Analytics and Statistics, Chang Gung Memorial Hospital at Linkou, Taoyuan, Taiwan; 5 Division of Urology, Department of Surgery, Chang Gung Memorial Hospital at Linkou, Chang Gung University College of Medicine, Taoyuan, Taiwan; 6 Division of Cardiology, Department of Internal Medicine, Chang Gung Memorial Hospital, Keelung, Taiwan; 7 Division of Hematology/Oncology, Department of Internal Medicine, Chang Gung Memorial Hospital at Linkou, Chang Gung University College of Medicine, Taoyuan, Taiwan; 8 Department of Public Health, College of Medicine, Chang Gung University, Taoyuan, Taiwan; 9 Biostatistics Core Laboratory, Molecular Medicine Research Center, Chang Gung University, Taoyuan, Taiwan; 10 Department of Oncology-Pathology, Karolinska Institutet, Stockholm, Sweden

## Abstract

**Importance:**

It is unclear whether the clinical benefits associated with non–vitamin K antagonist oral anticoagulants (NOACs) are similar to those associated with low-molecular-weight heparins (LMWHs) in Asian individuals with cancer and acute venous thromboembolism (VTE).

**Objective:**

To compare the risk of recurrent thromboembolic events and bleeding associated with use of a NOAC vs use of the LMWH enoxaparin in Asian individuals with cancer-associated VTE.

**Design, Setting, and Participants:**

This cohort study was conducted using data from the Chang Gung Research Database, a multi-institutional electronic medical records database in Taiwan. A cohort of 1109 patients with cancer-associated VTE were identified between January 1, 2012, and January 31, 2019. Data were analyzed from March 2019 through December 2020.

**Exposures:**

Receiving a NOAC (including rivaroxaban, apixaban, edoxaban, or dabigatran) or the LMWH enoxaparin.

**Main Outcomes and Measures:**

The primary outcomes were composite recurrent VTE or major bleeding. Stabilized inverse probability of treatment weighting was used to balance baseline covariates. We compared risks of recurrent VTE or major bleeding between groups using Cox proportional hazards models. In addition, we conducted an analysis using a Fine and Gray subdistribution hazard model that considered death as a competing risk.

**Results:**

Among 1109 patients with cancer and newly diagnosed VTE, 578 (52.1%) were women and the mean (SD) age at index date was 66.0 (13.0) years; 529 patients (47.7%) received NOACs and 580 patients (52.3%) received the LMWH enoxaparin. Composite recurrent VTE or major bleeding occurred in 75 patients (14.1%) in the NOAC group and 101 patients (17.4%) in the enoxaparin group (weighted hazard ratio [HR], 0.77; 95% CI, 0.56-1.07; *P* = .11). The groups had similar risk of VTE recurrence (HR, 0.62; 95% CI, 0.39-1.01; *P* = .05) and major bleeding (HR, 0.80; 95% CI, 0.52-1.24; *P* = .32) at 12 months of follow-up. However, taking a NOAC was associated with a significantly lower risk of gastrointestinal bleeding compared with receiving enoxaparin (10 patients [1.9%] vs 41 patients [7.1%]; HR, 0.29; 95% CI, 0.15-0.59; *P* < .001). Findings for both primary outcomes were consistent with competing risk analyses (recurrent VTE: HR, 0.68; 95% CI, 0.45-1.01; *P* = .05; major bleeding: HR, 0.77; 95% CI, 0.51-1.16; *P* = .21).

**Conclusions and Relevance:**

This cohort study found that in real-world practice, among Asian patients with cancer-associated VTE, use of a NOAC was associated with a similar risk for recurrent VTE or major bleeding compared with use of the LMWH enoxaparin. Nonetheless, use of a NOAC was associated with a significantly lower rate of gastrointestinal bleeding. Further prospective studies are needed to confirm these findings.

## Key Points

**Question:**

Are there differences in risks of venous thromboembolism (VTE) recurrence or of bleeding events associated with taking a non–vitamin K antagonist oral anticoagulant (NOAC) compared with receiving a low-molecular-weight heparin (LMWH) among Asian patients with cancer-associated VTE?

**Findings:**

In this cohort study of 1109 patients with cancer-associated VTE, use of a NOAC was not associated with increased risk of recurrent VTE or major bleeding but was associated with lower risk of gastrointestinal bleeding compared with use of the LMWH enoxaparin.

**Meaning:**

These findings suggest that in real-world practice, among Asian individuals with cancer-associated VTE, use of a NOAC had similar risk for recurrent VTE or major bleeding compared with use of LMWH.

## Teaser

This cohort study compares the risks of venous thromboembolism (VTE) recurrence in patients receiving non–vitamin K antagonist oral anticoagulants (NOACs) vs low-molecular-weight heparin in Asian individuals with cancer.

## Introduction

Venous thromboembolism (VTE), comprising pulmonary embolism (PE) and deep vein thrombosis (DVT), is a common cause of morbidity and the second leading cause of mortality in individuals with cancers after disease progression.^1^ Individuals with cancer have a 4-fold to 7-fold increased risk of VTE compared with the general population,^2,3,4^ corresponding to 1 VTE event out of 200 individuals with active cancer annually.^5^ Multiple factors are associated with risk of cancer-associated VTE, including tumor-associated factors (eg, cancer type and stage), treatment-associated factors (eg, major surgery, chemotherapy, antiangiogenic therapy, hormonal therapy, and central venous catheter use), and patient characteristics (eg, advanced age, obesity, and immobilization status).^6^

In the Randomized Comparison of Low-Molecular-Weight Heparin vs Oral Anticoagulant Therapy for the Prevention of Recurrent Venous Thromboembolism in Patients with Cancer (CLOT) clinical trial,^7^ use of the low-molecular-weight heparin (LMWH) dalteparin reduced risk of VTE recurrence by 52%, with similar rates for major bleeding events, compared with use of oral vitamin K antagonist (VKA). After the publication of the CLOT study results, use of LMWH for at least 6 months has been recommended over the last decade as the standard of care for the acute treatment and secondary prophylaxis of VTE in patients with cancer.^8,9^ However, continuous administration of LMWH over a course of several months is challenging because of its cost, inconvenience to patients, and adverse effects associated with subcutaneous injections, such as local pain and bruising.^10^

Over a decade ago, non–vitamin K antagonist oral anticoagulants (NOACs), including dabigatran, apixaban, edoxaban, and rivaroxaban, were introduced for the treatment of acute VTE. These medications were associated with favorable efficacy and safety profiles and were preferred over warfarin in clinical practice for patients with acute VTE. However, it remained inconclusive whether superior clinical benefits were associated with NOACs compared with LMWHs regarding acute VTE in patients with cancer, who have higher rates of recurrent VTE and major bleeding complications on anticoagulation agents compared with patients without cancer.^11,12^ Guidelines updated in 2020^13^ and 2019^14^ recommend use of edoxaban (Hokusai VTE cancer randomized clinical trial [RCT]^15^) and rivaroxaban (Anticoagulation Therapy in Selected Cancer Patients at Risk of Recurrence of Venous Thromboembolism [Select-D] RCT^16^) as alternatives to LMWHs for cancer-associated VET because of the acceptable results from the 2 RCTs. Moreover, a 2020 Caravaggio RCT^17^ demonstrated noninferior efficacy and comparable safety of apixaban compared with dalteparin. Nonetheless, these 3 RCTs have revealed somewhat conflicting results related to NOAC and LMWH comparisons, which might be partly because of characteristics used in patient selection (eg, cancer type, stage, and comorbidities).^18^ Furthermore, these 3 RCTs were not specifically designed to examine the effect of NOACs among Asian patients with cancer.

Therefore, because edoxaban, rivaroxaban, and apixaban have been considered the alternatives to LMWH for treating cancer-associated VTE, the effectiveness and safety associated with various NOACs need to be assessed in comparison with LMWHs in a real-world setting. To our knowledge, most observational studies to date compared the use of rivaroxaban with that of LMWHs, and few studies evaluated other NOACs.^19,20,21^ Therefore, we conducted this retrospective cohort study to assess the effectiveness and safety associated with edoxaban, rivaroxaban, apixaban, and dabigatran compared with a LMWH in treating acute VTE among Asian individuals with cancer.

## Methods

The ethics review board of Chang Gung Memorial Hospital (CGMH) system approved this cohort study and waived informed consent because patient data were deidentified before analysis. This study follows the Strengthening the Reporting of Observational Studies in Epidemiology (STROBE) reporting guideline.

### Data Sources

The study data were obtained from the Chang Gung Research Database (CGRD). This is an electronic medical records database that prospectively collects records of all emergency services use and inpatient and outpatient visits from the CGMH system. The CGMH system is multi-institutional, including 7 branches (ie, 4 tertiary academic medical centers and 3 teaching hospitals) across Taiwan. The hospital network contains 10 050 beds and treats 2.4 million patients every year, providing approximately 10% of the medical services used by the entire Taiwanese population.^22^ All patient data were anonymous and deidentified to protect personal privacy. A unique identification number for each patient was used for data linkage. The CGRD provided demographic characteristics, electronic medical records, pharmacy dispensing details, image reports, laboratory results, discharge summaries, and nursing records. Disease diagnoses and procedures were recorded using the *International Classification of Diseases, Ninth Revision, Clinical Modification* (*ICD-9-CM*)^23^ and *International Statistical Classification of Diseases, Tenth Revision, Clinical Modification* (*ICD-10-CM*)^24^ after 2016.

### Study Population

From January 1, 2012, to January 31, 2019, patients aged 18 years or older with active cancer who developed newly diagnosed VTE were identified. Newly diagnosed VTE was defined as the patient’s first time experiencing symptoms of VTE. The inclusive criteria for active cancer were taken from previous studies^15,16,17^ (eTable 1 in the Supplement). The diagnostic criteria for VTE included discharge diagnosis of DVT or PE per *ICD-9* or *ICD-10* codes (eTable 2 in the Supplement), radiographically confirmed cases using duplex ultrasonography for DVT or chest computed tomography (CT) scan and lung perfusion for PE, and outpatient diagnosis of DVT or PE at least twice with subsequent use of NOAC or LMWH. A flowchart of the study cohort enrollment process is illustrated in eFigure 1 in the Supplement. Moreover, to investigate the pure association, we excluded patients who had been treated with both NOAC and LMWH. However, according to Taiwan’s National Health Insurance regulations, patients need to have been on LMWH for at least 5 days before they can use edoxaban or dabigatran for VTE. Therefore, we placed patients with LMWH use within 14 days before NOAC use in the NOAC group.

### Exposure and Covariates

The NOACs used included dabigatran (Anatomical Therapeutic Chemical [ATC] classification system code, B01AE07), rivaroxaban (ATC code, B01AF01), apixaban (ATC code, B01AF02), and edoxaban (ATC code, B01AF03). Dosages were per Taiwan’s National Health Insurance regulation for treatment of VTE, namely 5 mg twice per day for apixaban, 60 mg once daily for edoxaban, 150 mg twice daily for dabigatran, and 15 mg twice for the first 21 days and then 20 mg per day for rivaroxaban. The LMWH considered for comparison in this study was enoxaparin (ATC code, B01AB05), which was the only LMWH available in CGMH. Considering the risk factors of VTE and bleeding, the potential confounders examined included demographic characteristics (ie, age and sex), baseline platelet and creatinine levels, Charlson comorbidity index, cancer type, tumor stage, active anticancer treatments, and risk factors associated with bleeding.

### Outcomes

The effectiveness end points included recurrent VTE or PE, which were confirmed based on new thrombus formation or a new blood vessel involved on duplex ultrasonography, chest CT scan, or lung perfusion. The safety end points of major bleeding were defined as the total hospitalized events of intracranial hemorrhage, major gastrointestinal (GI) bleeding, bleeding at other critical sites, and a decrease in hemoglobin of 2 g/dL or more over 24 hours. We defined the primary outcomes as the composite end points of effectiveness and safety. In addition, the secondary outcomes, including each end point, were measured. The diagnosis codes of CGRD were shifted from *ICD-9-CM* to *ICD-10-CM* after January 1, 2016. The safety outcomes were retrieved using data recorded in CGRD and *ICD-9-CM* or *ICD-10-CM* codes (eTable 2 in the Supplement).

### Study Design and Settings

All patients with cancer and newly diagnosed VTE who had received a NOAC were compared with patients who received LMWH treatment using a population-based cohort study design. The date of the first prescription of LMWH or NOAC was defined as the index date in the LMWH and NOAC groups. Study participants were followed up from the index date until the first occurrence of the outcomes, 1 year after the index date, death, or the end of follow-up (ie, January 2019), whichever came first. Furthermore, to reduce detection bias, patients with new events within 5 days after the index date were not included.

### Statistical Analysis

Patient demographic characteristics and covariates were presented and stratified by exposure group. Continuous variables were reported as mean (SD), and categorical data were presented as numbers and percentages. For confounding adjustment, all baseline covariates were included in the generalized boosting method model to calculate a propensity score. Stabilized inverse probability of treatment weighting was conducted to achieve covariate balance.^25^ The balance of potential confounders at baseline between the 2 exposure groups was estimated using standardized mean difference, with a value of 0.1 or less indicating an insignificant difference. We compared the risks of recurrent VTE and major bleeding between the groups using Cox proportional hazards models. The risks of time-to-event outcomes in the 2 groups were further compared using a Fine and Gray subdistribution hazard model that considered death as a competing risk. The LMWH group was used as the reference group.

A subgroup analysis was performed to determine whether the hazard ratios (HRs) of composite outcomes for the NOAC and LMWH groups were similar in the prespecified subgroups, which included age (ie, <65 years or ≥65 years), sex, platelet count (ie, <50 000, 50 000-100 000, or >100 000; to convert to ×10^9^ per liter, multiply by 1.0), hemoglobin level (ie, <8 g/dL, 8-10 g/dL, or >10 g/dL; to convert to grams per liter, multiply by 10.0), tumor type (ie, GI cancer or non-GI cancer), and cancer stage (ie, metastatic or nonmetastatic disease). The cumulative incidence of the composite outcomes for each NOAC group was compared using the log-rank test. To test the association of anticoagulant duration with the outcomes, we performed a stratification analysis by treatment duration of 3 months.

Statistical analysis was performed using SAS statistical software version 9.4 (SAS Institute). All statistical tests were 2-sided, and *P* < .05 was considered significant. Data were analyzed from March 2019 through December 2020.

## Results

### Baseline Clinical and Demographic Patient Characteristics

We identified 1109 patients with cancer and newly diagnosed VTE (578 [52.1%] women; mean (SD) age at index, 66.0 [13.0] years) who had initiated treatment with either a NOAC (529 patients [47.7%]) or an LMWH (580 patients [52.3%]) from January 1, 2012, to January 31, 2019 (eFigure 1 in the Supplement). Overall, 218 patients (19.7%) had GI tract cancer, including esophageal, GI, and colorectal cancer; 532 patients (48.0%) had hemoglobin levels of less than 10 g/dL and 183 patients (16.5%) had hemoglobin levels of less than 8 g/dL; and 242 (21.8%) patients had platelet counts of less than 100 000 per μl. Among patients receiving NOACs, 374 (70.7%) received rivaroxaban. Patients treated with a NOAC, compared with patients receiving an LMWH, were older (mean [SD] age at index date, 67.7 [13.3] years vs 64.6 [12.4] years; standardized mean difference, 0.24), more likely to have multiple cancers (92 patients [17.4%] vs 45 patients [7.8%]; standardized mean difference, 0.29), and less likely to have lower platelet levels (93 patients [17.6%] vs 59 patients [10.2%] with <50 000 platelets per μl; standardized mean difference, 0.21) or metastatic disease (211 patients [39.9%] vs 325 patients [56.0%]; standardized mean difference, 0.32) or currently be receiving chemotherapy (239 patients [45.2%] vs 371 patients [64.0%]; standardized mean difference, 0.38) or radiotherapy (129 patients [24.4%] vs 202 patients [34.8%]; standardized mean difference, 0.22). No significant intergroup difference was found for sex, creatinine level, Charlson comorbidity index, tumor type, or risk of bleeding (Table 1).

**Table 1.** Baseline Demographic and Clinical Characteristics

Characteristic	Total, No. (%) (N = 1109)	Before propensity score weighting^a^			After propensity score weighting^a^		
		NOAC, No. (%) (n = 529)	LMWH, No. (%) (n = 580)	Standardized mean difference^b^	NOAC, No. (%) (n = 468)	LMWH, No. (%) (n = 525)	Standardized mean difference^b^
Women	578 (52.1)	283 (53.5)	295 (50.9)	0.05	247 (52.8)	270 (51.4)	0.02
Men	531 (47.9)	246 (46.5)	285 (49.1)	0.05	221 (47.2)	256 (48.6)	0.02
Age at index date, mean (SD), y	66.0 (13.0)	67.7 (13.3)	64.6 (12.4)	0.24	66.5 (12.3)	65.8 (12.0)	0.05
18-49	129 (11.6)	49 (9.3)	80 (13.8)	0.14	50 (10.6)	66 (12.6)	0.06
50-64	403 (36.3)	171 (32.3)	232 (40.0)	0.16	165 (35.2)	192 (36.5)	0.02
65-74	277 (25.0)	132 (25.0)	145 (25.0)	<0.001	119 (25.5)	133 (25.3)	<0.001
75-85	239 (21.6)	140 (26.5)	99 (17.1)	0.22	108 (23.1)	109 (20.7)	0.05
≥85	61 (5.5)	37 (7.0)	24 (4.1)	0.12	26 (5.6)	26 (4.9)	0.03
Thromboembolism type							
PE and DVT	38 (3.4)	26 (4.9)	12 (2.1)	0.15	17 (3.6)	12 (2.3)	0.07
PE only	461 (41.6)	191 (36.1)	270 (46.6)	0.21	179 (38.3)	225 (42.8)	0.09
DVT only	610 (55.0)	312 (59.0)	298 (51.4)	0.15	272 (58.1)	289 (54.9)	0.06
Serum creatinine, mean (SD), mg/dL	1.0 (1.2)	1.0 (1.2)	1.1 (1.3)	0.08	1.0 (1.4)	1.0 (1.0)	<0.001
Platelet count, mean (SD), No. ×10^3^/μl	224.5 (112.6)	237.2 (116.0)	214.1 (108.7)	0.20	226.1 (106.9)	218.8 (99.3)	0.07
Platelet count, per μl							
>100 000	867 (78.2)	405 (76.6)	462 (79.7)	0.07	366 (78.3)	409 (77.8)	0.01
50 000-100 000	90 (8.1)	31 (5.9)	59 (10.2)	0.15	36 (7.7)	44 (8.5)	0.02
<50 000	152 (13.7)	93 (17.6)	59 (10.2)	0.21	66 (14.1)	72 (13.8)	<0.001
Hemoglobin level, g/dL							
Mean (SD)	10.6 (1.9)	10.8 (1.9)	10.4 (1.8)	0.21	10.6 (1.8)	10.5 (1.7)	0.05
>10	577 (52.0)	278 (52.6)	299 (51.6)	0.02	238 (50.9)	270 (51.3)	0.008
8-10	349 (31.5)	142 (26.8)	207 (35.7)	0.19	145 (31.0)	170 (32.3)	0.02
<8	183 (16.5)	109 (20.6)	74 (12.8)	0.21	85 (18.1)	86 (16.4)	0.05
Charlson Comorbidity Index score							
0	51 (4.6)	23 (4.3)	28 (4.8)	0.02	16 (3.5)	24 (4.5)	0.05
1-2	145 (13.1)	89 (16.8)	56 (9.7)	0.21	66 (14.2)	62 (11.8)	0.07
≥3	913 (82.3)	417 (78.8)	496 (85.5)	0.17	385 (82.3)	440 (83.7)	0.03
Current cancer treatment							
Chemotherapy	610 (55.0)	239 (45.2)	371 (63.9)	0.38	253 (54.1)	307 (58.4)	0.08
Radiotherapy	331 (29.8)	129 (24.4)	202 (34.8)	0.22	128 (27.4)	164 (31.1)	0.08
Chemotherapy or radiotherapy	707 (63.8)	280 (52.9)	427 (73.6)	0.44	287 (61.4)	353 (67.2)	0.12
Tumor type							
Brain	7 (0.6)	5 (0.9)	2 (0.3)	0.07	3 (0.7)	1 (0.3)	0.05
Breast	56 (5.0)	27 (5.1)	29 (5.0)	0.005	23 (4.9)	27 (5.2)	0.01
Colorectal	151 (13.6)	79 (14.9)	72 (12.4)	0.07	64 (13.7)	71 (13.5)	0.006
Esophagus	17 (1.5)	8 (1.5)	9 (1.6)	0.008	7 (1.4)	8 (1.5)	0.008
Gallbladder	38 (3.4)	11 (2.1)	27 (4.7)	0.14	14 (2.4)	19 (3.7)	0.03
Genitourinary^c^	75 (6.8)	29 (5.5)	46 (7.9)	0.09	29 (6.1)	37 (7.0)	0.03
Gynecologic	72 (6.5)	40 (7.6)	32 (5.5)	0.08	30 (6.5)	30 (5.7)	0.03
Intestine	4 (0.4)	1 (0.2)	3 (0.5)	0.05	1 (0.2)	2 (0.4)	0.03
Lung	210 (18.9)	106 (20.0)	104 (17.9)	0.05	98 (20.9)	102 (19.4)	0.04
Myeloma	1 (0.1)	1 (0.2)	0	0.06	1 (0.1)	0	0.04
Other	183 (16.5)	75 (14.2)	108 (18.6)	0.11	76 (16.3)	90 (17.2)	0.02
Ovarian	26 (2.3)	10 (1.9)	16 (2.8)	0.05	11 (2.3)	12 (2.4)	0.007
Pancreatic	46 (4.1)	14 (2.6)	32 (5.5)	0.14	18 (3.9)	23 (4.4)	0.02
Prostate	40 (3.6)	23 (4.3)	17 (2.9)	0.07	18 (3.8)	18 (3.4)	0.02
Stomach	46 (4.1)	8 (1.5)	38 (6.6)	0.26	12 (2.5)	24 (4.6)	0.11
Multiple	137 (12.4)	92 (17.4)	45 (7.8)	0.29	64 (13.8)	60 (11.5)	0.06
Stage of cancer							
Early or locally advanced	460 (41.5)	240 (45.4)	220 (37.9)	0.15	198 (42.4)	212 (40.3)	0.04
Metastatic	536 (48.3)	211 (39.9)	325 (56.0)	0.32	217 (46.5)	271 (51.6)	0.10
Hematologic	25 (2.3)	15 (2.8)	10 (1.7)	0.07	11 (2.3)	13 (2.4)	0.007
Undetermined	88 (7.9)	63 (11.9)	25 (4.3)	0.28	41 (8.9)	30 (5.7)	0.12
Risk factors for bleeding, No.^d^							
0	67 (6.0)	42 (7.9)	25 (4.3)	0.15	28 (5.9)	30 (5.8)	0.004
1	622 (56.1)	305 (57.7)	317 (54.7)	0.06	278 (59.5)	296 (56.4)	0.06
2	330 (29.8)	140 (26.5)	190 (32.8)	0.13	125 (26.7)	153 (29.2)	0.05
≥3	90 (8.1)	42 (7.9)	48 (8.3)	0.01	37 (7.9)	46 (8.7)	0.02

Abbreviations: DVT, deep vein thrombosis; LMWH, low-molecular-weight heparin; NOAC, non–vitamin K antagonist oral anticoagulant agent; PE, pulmonary embolism.

SI conversion factors: To convert creatinine to micromoles per liter, multiply by 88.4; hemoglobin to grams per liter, multiply by 10.0; and platelet count to ×10^9^ per liter, multiply by 1.0.

^a^ All covariates listed were used to calculate the propensity score.

^b^ An absolute standardized mean difference of 0.1 or less indicated a negligible difference.

^c^ Included renal, bladder, ureteral, and testicular cancers but not prostate cancer.

^d^ Included surgery within 2 weeks before index date, use of antiplatelet agents, primary or metastatic brain tumor, regionally advanced or metastatic cancer, gastrointestinal or urothelial cancer, and treatment with bevacizumab within the 6-week period before randomization.

We included patients with low hemoglobin and platelet levels, who were not eligible for randomized trials.^15,16,17^ There were 251 patients (47.4%) in the NOAC group and 281 patients (48.4%) in the LMWH group with hemoglobin levels of 10 g/dL or lower. The NOAC group had 374 patients (70.7%) taking rivaroxaban, 51 patients (9.6%) taking apixaban, 35 patients (6.6%) taking edoxaban, and 11 patients (2.1%) taking dabigatran. There were 58 patients (11%) who had received more than one kind of NOAC. After inverse probability of treatment weighting, no statistically significant intergroup difference was observed regarding demographic characteristics, tumor type, or risk factor for bleeding at baseline (Table 1).

### Effectiveness and Safety Outcomes

**Figure 1.**
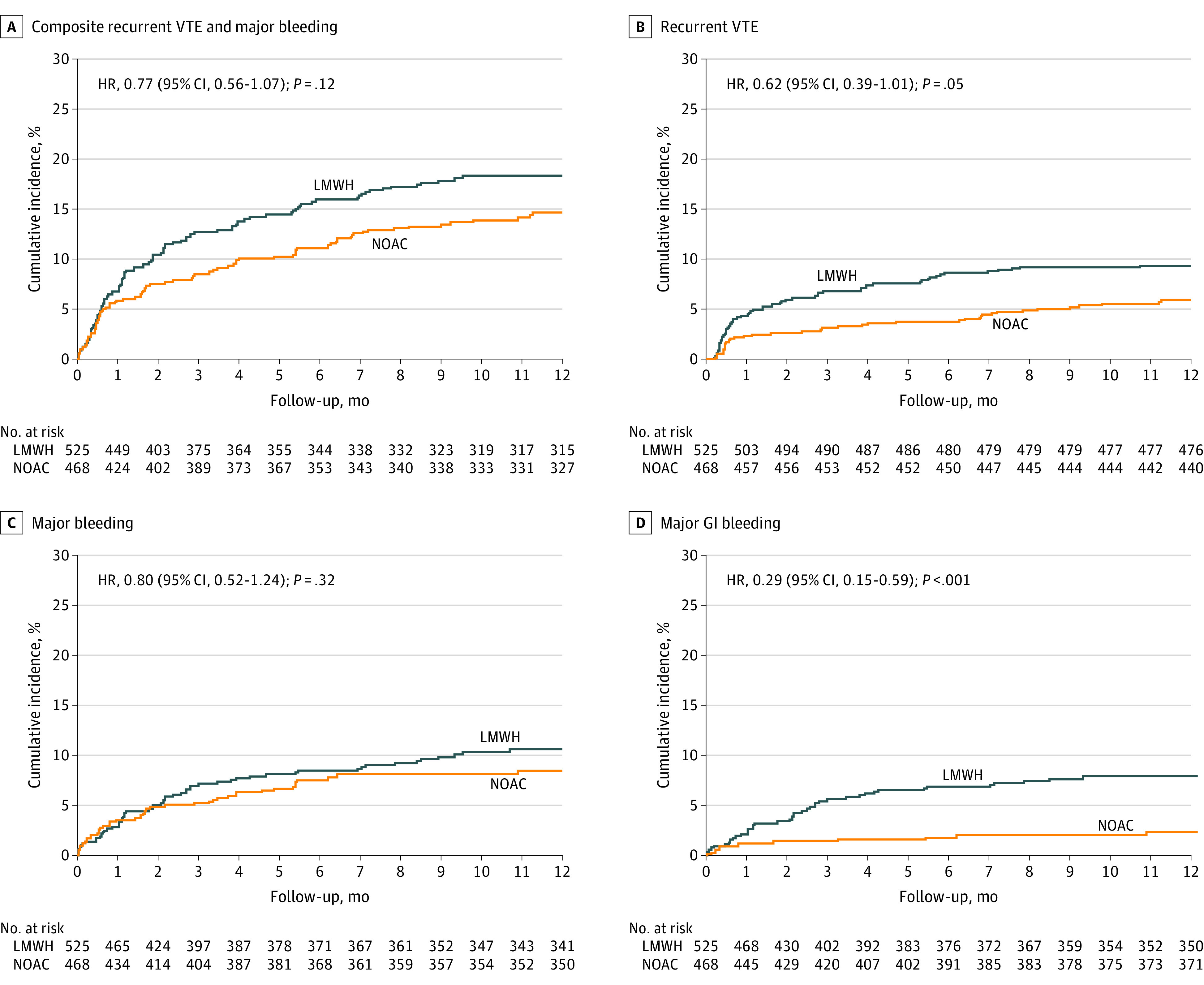
Cumulative Probability of Event Rates

Effectiveness and safety outcomes are provided in Table 2. The cumulative incidences of outcomes are plotted in Figure 1. There were 38 patients (7.2%) in the NOAC group and 60 patients (10.3%) in the LMWH group who met the effectiveness outcome of recurrent VTE, for a similar risk (HR, 0.62; 95% CI, 0.39-1.01; *P* = .05). Recurrent VTE or major bleeding occurred in 75 patients (14.1%) treated with NOAC and 101 patients (17.4%) treated with LMWH within 1 year (HR, 0.72; 95% CI, 0.53-0.97; *P* = .02). Overall, the weighted HR was 0.77 (95% CI, 0.56-1.07; *P* = .11), indicating no significant intergroup difference in composed end points. While recurrent VTE rates were lower in the NOAC group, the weighted HRs did not exhibit a significant difference. There were 39 patients (7.4%) in the NOAC group and 55 patients (9.5%) in the LMWH group with major bleeding events, so rates were similar (weighted HR, 0.80; 95% CI, 0.52-1.24; *P* = .32). We observed significantly lower GI bleeding rates in the NOAC group compared with the LMWH group (10 patients [1.9%.] vs 41 patients [7.1%]; weighted HR, 0.29; 95% CI, 0.15-0.59; *P* < .001). Patients treated with NOAC had lower mortality rates than patients treated with LMWH (Table 2). However, the results of effectiveness, safety, and combined outcomes after adjusting competing risks remained consistent with the primary analyses (recurrent VTE: HR, 0.68; 95% CI, 0.45-1.01; *P* = .05; major bleeding: HR, 0.77; 95% CI, 0.51-1.16; *P* = .21).

**Table 2.** Effectiveness and Safety Clinical Outcomes

Outcome	No. (%)		Unweighted		Weighted		Competing risk	
	NOAC (n = 529)	LMWH (n = 580)	HR (95% CI)	*P* value	HR (95% CI)	*P* value	SHR (95% CI)	*P* value
Composite effectiveness and safety outcome								
Recurrent VTE or major bleeding	75 (14.1)	101 (17.4)	0.72 (0.53-0.97)	.02	0.77 (0.56-1.07)	.11	0.79 (0.59-1.07)	.12
Effectiveness outcome, recurrent								
VTE	38 (7.2)	60 (10.3)	0.68 (0.45-1.01)	.05	0.62 (0.39-1.01)	.05	0.68 (0.45-1.01)	.05
DVT	22 (4.2)	27 (4.7)	0.88 (0.50-1.55)	.66	0.77 (0.41-1.44)	.40	0.88 (0.50-1.55)	.66
PE	16 (3.0)	33 (5.7)	0.52 (0.29-0.95)	.03	0.50 (0.25-1.01)	.05	0.52 (0.29-0.95)	.03
Safety outcome								
Bleeding								
Major^a^	39 (7.4)	55 (9.5)	0.69 (0.46-1.04)	.07	0.80 (0.52-1.24)	.32	0.77 (0.51-1.16)	.21
Major GI	10 (1.9)	41 (7.1)	0.23 (0.12-0.47)	<.001	0.29 (0.15-0.59)	<.001	0.26 (0.13-0.52)	<.001
Non-GI	29 (5.5)	18 (3.1)	1.61 (0.90-2.90)	.11	1.84 (0.99-3.45)	.05	1.79 (1.00-3.23)	.05
All-cause mortality	82 (15.5)	186 (32.1)	0.43 (0.33-0.55)	<.001	0.61 (0.47-0.80)	<.001	NA	NA

Abbreviations: DVT, deep vein thrombosis; GI, gastrointestinal; HR, hazard ratio; LMWH, low-molecular-weight heparin; NA, not applicable; NOAC, non–vitamin K antagonist oral anticoagulant agent; PE, pulmonary embolism; SHR, subdistribution hazard ratio; VTE, venous thromboembolism.

^a^ For patients who had more than 1 event, only the first event was counted. Major bleeding included major GI bleeding, intracranial hemorrhage, bleeding at other critical sites, and decrease in hemoglobin of 2 g/dL or more over 24 hours.

### Subgroup Analyses and Risk per NOAC Type

**Figure 2.**
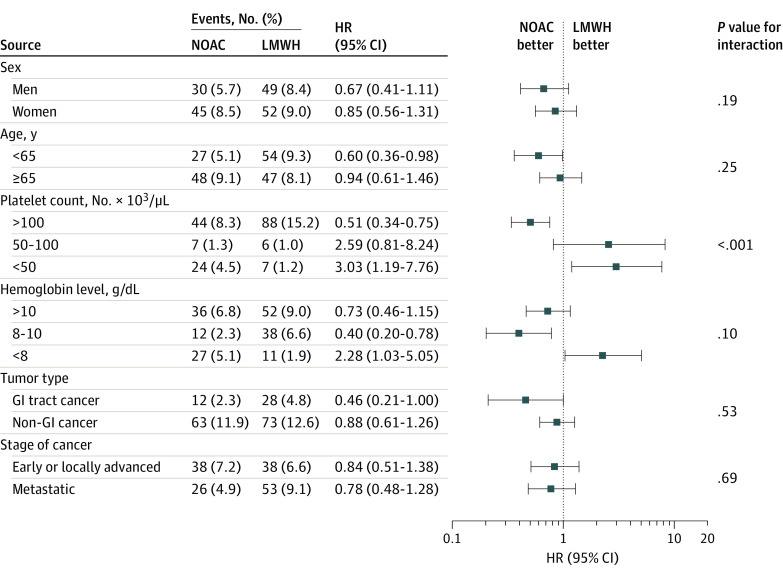
Composite Outcomes of Recurrent Venous Thromboembolism (VTE) and Major Bleeding by Subgroup NOAC indicates non–vitamin K antagonist oral anticoagulant; LMWH, low-molecular-weight heparin; GI, gastrointestinal; HR, hazard ratio. To convert hemoglobin to grams per liter, multiply by 10.0; and platelet count to ×10^9^ per liter, multiply by 1.0.

**Figure 3.**
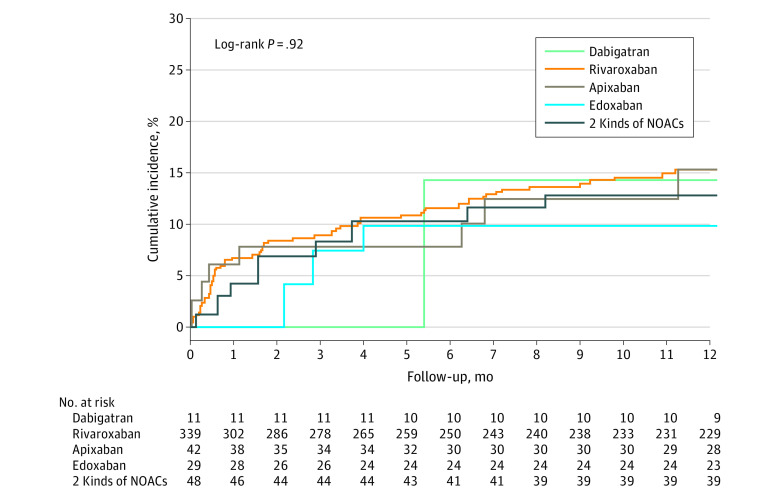
Cumulative Incidence of Recurrent VTE or Major Bleeding at 1-Year Follow-up by NOAC Subtype VTE indicates venous thromboembolism; NOAC, non–vitamin K antagonist oral anticoagulant.

In subgroup analysis, all risks for combined recurrent VTE and major bleeding remained constant across all planned subgroups except platelet count (Figure 2). Patients with platelet counts of less than 50 000 per μl treated with NOACs had a higher risk of recurrent VTE (15 patients [2.8%] vs 4 patients [0.7%]; *P* for interaction <.001) (eFigure 2 in the Supplement) and composite VTE and major bleeding (24 patients [4.5%] vs 7 patients [1.2%]; HR, 3.03; 95% CI, 1.19-7.76; *P* for interaction <.001) (Figure 2). For safety outcomes of major bleeding or GI bleeding, the results were consistent across all subgroups (eFigures 3 and 4 in the Supplement). In the stratified analysis by anticoagulant duration, we found consistent results regardless of treatment duration (ie, less 3 months or 3 months or more) (eFigure 5 in the Supplement). No significant differences were found in the cumulative incidence of recurrent VTE or major bleeding by NOAC subtype (Figure 3).

## Discussion

To our knowledge, this is the first real-world cohort study that compared the effectiveness and safety associated with 4 different types of NOAC with LMWH in Asian patients with cancer-associated VTE. This study found that treatment with NOAC was associated with similar rates of composite outcomes of recurrent VTE or major bleeding compared with treatment with the LMWH enoxaparin. The effectiveness outcome of recurrent VTE was similar between the NOAC and enoxaparin groups. The recurrent VTE rates associated with NOAC treatment were consistent with the evidence of the efficacy associated with NOACs in the treatment of VTE in such patients.^17^ Moreover, the safety outcome of major bleeding was similar between the NOAC and enoxaparin groups. The observed numerical intergroup difference regarding major bleeding was primarily associated with the significantly lower rate of major GI bleeding with NOAC use compared with enoxaparin use. The results for effectiveness and safety outcomes remained consistent with the primary analyses after adjusting for death as competing risk, providing further evidence for the robustness of the main findings.

The rate of major bleeding was similar between NOAC and enoxaparin groups, which was in contrast to the results of a 2018 randomized clinical trial^15^ and a 2019 meta-analysis,^26^ which found higher incidences of bleeding with use of other NOACs compared with use of dalteparin among patients with cancer and VTE.^15,26^ Furthermore, we determined a significantly lower rate of major GI bleeding events among patients treated with NOAC compared with those treated with enoxaparin. This finding is in contrast to the results of 2 randomized clinical trials from 2018^15,16^ that noted numerically higher rates of GI bleeding with edoxaban or rivaroxaban compared with LMWH in cancer-associated VTE. These studies found an increase in risk of GI bleeding in patients with GI cancer, and guidelines were created^14,27^ suggesting caution in the use of edoxaban or rivaroxaban in patients with GI cancer and VTE. In our study, GI tract cancer, including esophageal, GI, and colorectal cancer, accounted for 19.7% of the population, which was comparable with the 20.7% in the Hokusai VTE cancer study^15^ and 24.9% in the Caravaggio trial.^17^ However, we did not detect differences associated with major bleeding or major GI bleeding between patients with GI tract cancer and those with non-GI tract cancer.

The exact mechanism associated with the discrepancy of GI bleeding outcome between our results and the previous study is unclear, but one potential explanation could be racial/ethnic differences between study populations. Unlike the studies^15,16,17^ that included non-Asian patients with cancer, our study focused on the East Asian population from Taiwan. Of note, it has been suggested that the reduction in critical organ bleeding associated with use of rivaroxaban vs warfarin is significantly greater in the East Asian population compared with the non-East Asian population.^28^ A meta-analysis by Wang et al^29^ found that the improved safety outcomes associated with use of standard-dose NOACs, including dabigatran and edoxaban, compared with use of vitamin K antagonists was greater among Asians compared with non-Asians with GI bleeding (odds ratio [OR], 0.79; 95% CI, 0.48-1.32 vs OR, 1.44; 95% CI, 1.12-1.85; *P* for interaction = 0.04). Therefore, our findings suggest that racial/ethnic differences may be associated with GI bleeding in treatment with NOACs vs LMWH in cancer-associated VTE. Nonetheless, further prospective studies are warranted to investigate these results.

The strengths of our study are the inclusion of a large variety of cancer types, with approximately one-third of the population having lung or colorectal cancer, which are associated with high thromboembolic risk.^30^ The cancer distribution in our study was consistent with rates reported in previous studies involving cancer patients with VTE. Nonetheless, unlike the Select-D trial,^16^ which excluded patients with baseline hemoglobin levels of less than 10 g/dL, and the Caravaggio trial,^17^ which excluded patients with baseline hemoglobin levels of less than 8 g/dL, 48% of our study population had hemoglobin levels less than 10 g/dL and 16.5% had hemoglobin levels less than 8 g/dL. Approximately one-fifth of our patient population had platelet counts less than 100 000 per μl, and patients with this characteristic were excluded from the Select-D trial.^16^ Although our analysis included a population with a wide range of characteristics, which was more applicable to real-world clinical practice, the subgroup analysis found that use of NOACs was associated with significantly increased recurrent VTE and major bleeding in patients with platelet levels of less than 50 000 /ul. These findings could help clinicians determine individualized anticoagulation strategies.

### Limitations

This study has several limitations. First, although we included 4 types of NOACs, including rivaroxaban, apixaban, edoxaban, and dabigatran, most patients (70.7%) in the NOAC group received rivaroxaban. However, we did not detect any significant difference regarding composite recurrent VTE or major bleeding among the 4 different types of NOACs. Second, because of the retrospective nature of the study, the 2 groups may have had inherent differences. To reduce selection bias, we used propensity score weighting to balance differences associated with major characteristics at baseline. Furthermore, the results of effectiveness or safety or combined outcomes after adjusting for competing risks remained consistent with the primary analyses. Third, information on prescribed drugs may not reflect actual use. Therefore, an underestimation associated with noncompliance is likely.

## Conclusions

This cohort study found that NOAC therapy, compared with LMWH therapy, in Asian patients with cancer was associated with similar rates of recurrent VTE, with no increase in major bleeding events. Furthermore, a significant decrease in GI bleeding risk was observed with NOACs. These results suggest that NOACs are associated with effective and safe outcomes as alternatives to LMWHs for the treatment of cancer-associated VTE in Asian patients in real-world practice.

## Notes

Supplement. **eTable 1.** Inclusive Criteria Defining Active Cancer in the Study Cohort **eTable 2.** Codes Used to Define the Cancer Population and Safety Outcomes in the Study Cohort **eFigure 1.** Enrollment and Follow-Up of Study Patients **eFigure 2.** Analysis for Subgroups for Outcomes of Recurrent VTE for Patients Treated With NOAC or LMWH **eFigure 3.** Analysis for Subgroups for Outcomes of Major Bleeding for Patients Treated With NOAC or LMWH **eFigure 4.** Analysis for Subgroups for Outcomes of Major GI Bleeding for Patients Treated With NOAC or LMWH **eFigure 5.** Stratified Analysis by Anticoagulant Duration

## References

[1] Khorana AA, Francis CW, Culakova E, Kuderer NM, Lyman GH Thromboembolism is a leading cause of death in cancer patients receiving outpatient chemotherapy. J Thromb Haemost. 2007;5(3):632-634. doi:10.1111/j.1538-7836.2007.02374.x 17319909

[2] Timp JF, Braekkan SK, Versteeg HH, Cannegieter SC Epidemiology of cancer-associated venous thrombosis. Blood. 2013;122(10):1712-1723. doi:10.1182/blood-2013-04-460121 23908465

[3] Heit JA, Spencer FA, White RH The epidemiology of venous thromboembolism. J Thromb Thrombolysis. 2016;41(1):3-14. doi:10.1007/s11239-015-1311-6 26780736 PMC4715842

[4] Blom JW, Doggen CJ, Osanto S, Rosendaal FR Malignancies, prothrombotic mutations, and the risk of venous thrombosis. JAMA. 2005;293(6):715-722. doi:10.1001/jama.293.6.715 15701913

[5] Lee AY, Levine MN Venous thromboembolism and cancer: risks and outcomes. Circulation. 2003;107(23)(suppl 1):I17-I21. doi:10.1161/01.CIR.0000078466.72504.AC 12814981

[6] Young A, Chapman O, Connor C, Poole C, Rose P, Kakkar AK Thrombosis and cancer. Nat Rev Clin Oncol. 2012;9(8):437-449. doi:10.1038/nrclinonc.2012.106 22777060

[7] Lee AY, Levine MN, Baker RI, ; Randomized Comparison of Low-Molecular-Weight Heparin versus Oral Anticoagulant Therapy for the Prevention of Recurrent Venous Thromboembolism in Patients with Cancer (CLOT) Investigators Low-molecular-weight heparin versus a coumarin for the prevention of recurrent venous thromboembolism in patients with cancer. N Engl J Med. 2003;349(2):146-153. doi:10.1056/NEJMoa025313 12853587

[8] Mandalà M, Falanga A, Roila F; ESMO Guidelines Working Group Management of venous thromboembolism (VTE) in cancer patients: ESMO clinical practice guidelines. Ann Oncol. 2011;22(suppl 6):vi85-vi92. doi:10.1093/annonc/mdr392 21908511

[9] Lyman GH, Khorana AA, Kuderer NM, ; American Society of Clinical Oncology Clinical Practice Venous thromboembolism prophylaxis and treatment in patients with cancer: American Society of Clinical Oncology clinical practice guideline update. J Clin Oncol. 2013;31(17):2189-2204. doi:10.1200/JCO.2013.49.1118 23669224

[10] Mahé I, Chidiac J, Helfer H, Noble S Factors influencing adherence to clinical guidelines in the management of cancer-associated thrombosis. J Thromb Haemost. 2016;14(11):2107-2113. doi:10.1111/jth.13483 27566698

[11] Prandoni P, Lensing AW, Piccioli A, Recurrent venous thromboembolism and bleeding complications during anticoagulant treatment in patients with cancer and venous thrombosis. Blood. 2002;100(10):3484-3488. doi:10.1182/blood-2002-01-0108 12393647

[12] Hutten BA, Prins MH, Gent M, Ginsberg J, Tijssen JG, Büller HR Incidence of recurrent thromboembolic and bleeding complications among patients with venous thromboembolism in relation to both malignancy and achieved international normalized ratio: a retrospective analysis. J Clin Oncol. 2000;18(17):3078-3083. doi:10.1200/JCO.2000.18.17.3078 10963635

[13] Key NS, Khorana AA, Kuderer NM, Venous thromboembolism prophylaxis and treatment in patients with cancer: ASCO clinical practice guideline update. J Clin Oncol. 2020;38(5):496-520. doi:10.1200/JCO.19.01461 31381464

[14] Farge D, Frere C, Connors JM, ; International Initiative on Thrombosis and Cancer (ITAC) advisory panel 2019 international clinical practice guidelines for the treatment and prophylaxis of venous thromboembolism in patients with cancer. Lancet Oncol. 2019;20(10):e566-e581. doi:10.1016/S1470-2045(19)30336-5 31492632

[15] Raskob GE, van Es N, Verhamme P, ; Hokusai VTE Cancer Investigators Edoxaban for the treatment of cancer-associated venous thromboembolism. N Engl J Med. 2018;378(7):615-624. doi:10.1056/NEJMoa1711948 29231094

[16] Young AM, Marshall A, Thirlwall J, Comparison of an oral factor Xa inhibitor with low molecular weight heparin in patients with cancer with venous thromboembolism: results of a randomized trial (Select-D). J Clin Oncol. 2018;36(20):2017-2023. doi:10.1200/JCO.2018.78.8034 29746227

[17] Agnelli G, Becattini C, Meyer G, ; Caravaggio Investigators Apixaban for the treatment of venous thromboembolism associated with cancer. N Engl J Med. 2020;382(17):1599-1607. doi:10.1056/NEJMoa1915103 32223112

[18] Lee AYY Anticoagulant therapy for venous thromboembolism in cancer. N Engl J Med. 2020;382(17):1650-1652. doi:10.1056/NEJMe2004220 32223115

[19] Phelps MK, Wiczer TE, Erdeljac HP, A single center retrospective cohort study comparing low-molecular-weight heparins to direct oral anticoagulants for the treatment of venous thromboembolism in patients with cancer — a real world experience. J Oncol Pharm Pract. 2019;25(4):793-800. doi:10.1177/1078155218757856 29460705

[20] Pritchard ER, Murillo JR, Putney D, Hobaugh EC Single-center, retrospective evaluation of safety and efficacy of direct oral anticoagulants versus low-molecular-weight heparin and vitamin K antagonist in patients with cancer. J Oncol Pharm Pract. 2019;25(1):52-59. doi:10.1177/1078155217726158 28825375

[21] Alzghari SK, Seago SE, Garza JE, Retrospective comparison of low molecular weight heparin vs. warfarin vs. oral Xa inhibitors for the prevention of recurrent venous thromboembolism in oncology patients: the Re-CLOT study. J Oncol Pharm Pract. 2018;24(7):494-500. doi:10.1177/1078155217718382 28714376

[22] Chang Gung Memorial Hospital (2019) About us: overview. Chang Gung Medical Foundation. Accessed January 14, 2020. http://www.chang-gung.com/en/about.aspx?id=11&bid=1

[23] Centers for Disease Control and Prevention *International Classification of Diseases, Ninth Revision, Clinical Modification (ICD-9-CM)* Accessed December 22, 2020. https://www.cdc.gov/nchs/icd/icd9cm.htm

[24] Centers for Disease Control and Prevention *International Classification of Diseases, Tenth Revision, Clinical Modification (ICD-10-CM)* Accessed December 22, 2020. https://www.cdc.gov/nchs/icd/icd10cm.htm

[25] Xu S, Ross C, Raebel MA, Shetterly S, Blanchette C, Smith D Use of stabilized inverse propensity scores as weights to directly estimate relative risk and its confidence intervals. Value Health. 2010;13(2):273-277. doi:10.1111/j.1524-4733.2009.00671.x 19912596 PMC4351790

[26] Kirkilesis GI, Kakkos SK, Tsolakis IA Editor’s choice: a systematic review and meta-analysis of the efficacy and safety of anticoagulation in the treatment of venous thromboembolism in patients with cancer. Eur J Vasc Endovasc Surg. 2019;57(5):685-701. doi:10.1016/j.ejvs.2018.11.004 31097186

[27] Konstantinides SV, Meyer G, Becattini C, ; ESC Scientific Document Group 2019 ESC guidelines for the diagnosis and management of acute pulmonary embolism developed in collaboration with the European Respiratory Society (ERS). Eur Heart J. 2020;41(4):543-603. doi:10.1093/eurheartj/ehz405 31504429

[28] Wong KS, Hu DY, Oomman A, ; Executive Steering Committee and the ROCKET AF Study Investigators Rivaroxaban for stroke prevention in East Asian patients from the ROCKET AF trial. Stroke. 2014;45(6):1739-1747. doi:10.1161/STROKEAHA.113.002968 24763930

[29] Wang KL, Lip GY, Lin SJ, Chiang CE, Non-Vitamin K Non-vitamin k antagonist oral anticoagulants for stroke prevention in Asian patients with nonvalvular atrial fibrillation: meta-analysis. Stroke. 2015;46(9):2555-2561. doi:10.1161/STROKEAHA.115.009947 26304863 PMC4542566

[30] Mahé I, Chidiac J, Bertoletti L, ; RIETE investigators The clinical course of venous thromboembolism may differ according to cancer site. Am J Med. 2017;130(3):337-347. doi:10.1016/j.amjmed.2016.10.017 27884650

