# Supplementary material for: Comparison Between Non–vitamin K Antagonist Oral Anticoagulants and Low-Molecular-Weight Heparin in Asian Individuals With Cancer-Associated Venous Thromboembolism
Source: JAMA Netw Open. 2021 Feb 3;4(2):e2036304. doi: 10.1001/jamanetworkopen.2020.36304 (PMC7859846; doi:10.1001/jamanetworkopen.2020.36304)
Supplement: Supplement. — eTable 1. Inclusive Criteria Defining Active Cancer in the Study Cohort eTable 2. Codes Used to Define the Cancer Population and Safety Outcomes in the Study Cohort eFigure 1. Enrollment and Follow-Up of Study Patients eFigure 2. Analysis for Subgroups for Outcomes of Recurrent VTE for Patients Treated With NOAC or LMWH eFigure 3. Analysis for Subgroups for Outcomes of Major Bleeding for Patients Treated With NOAC or LMWH eFigure 4. Analysis for Subgroups for Outcomes of Major GI Bleeding for Patients Treated With NOAC or LMWH eFigure 5. Stratified Analysis by Anticoagulant Duration [file jamanetwopen-e2036304-s001.pdf]

Supplemental Online Content

Chen DY, Tseng CN, Hsieh MJ, et al. Comparison between non–vitamin K antagonist oral anticoagulants and low-molecular-weight heparin in Asian individuals with cancer-associated venous thromboembolism. *JAMA Netw Open*. 2021;4(2):e2036304. doi:10.1001/jamanetworkopen.2020.36304

eTable 1. Inclusive Criteria Defining Active Cancer in the Study Cohort
eTable 2. Codes Used to Define the Cancer Population and Safety Outcomes in the Study Cohort
eFigure 1. Enrollment and Follow-Up of Study Patients
eFigure 2. Analysis for Subgroups for Outcomes of Recurrent VTE for Patients Treated With NOAC or LMWH
eFigure 3. Analysis for Subgroups for Outcomes of Major Bleeding for Patients Treated With NOAC or LMWH
eFigure 4. Analysis for Subgroups for Outcomes of Major GI Bleeding for Patients Treated With NOAC or LMWH
eFigure 5. Stratified Analysis by Anticoagulant Duration

This supplemental material has been provided by the authors to give readers additional information about their work.

© 2021 Chen DY et al. *JAMA Network Open.*

eTable1. Inclusive Criteria Defining Active Cancer in the Study Cohort

Cancer diagnosis within 6 months before index date
Metastatic cancer
Hematology cancer
Patients treated with radiotherapy or systemic therapy
Patients with consecutive oncology outpatient visits more than twice within one yea

© 2021 Chen DY et al. *JAMA Network Open.*

eTable 2. Codes Used to Define the Cancer Population and Safety Outcomes in the Study Cohort

	**International Classification of Disease 9th revision (ICD-9) code**	**International Classification of Disease 10th revision (ICD-10) code**	**Diagnostic Definition**
**Cancer**	140-209	C00-C80, C7A, C7B, C81-C96	
**Basal cell carcinoma**	173.01, 173.11, 173.21, 173.31, 173.41, 173.51, 173.61, 173.71, 173.81, 173.91	C44.01, C44.11, C44.21, C44.31, C44.41, C44.51, C44.61, C44.71, C44.81, C44.91	
**Squamous cell skin cancer**	173.02, 173.12, 173.22, 173.32, 173.42, 173.52, 173.62, 173.72, 173.82, 173.92	C44.02, C44.12, C44.22, C44.32, C44.42, C44.52, C44.62, C44.72, C44.82, C44.92	
**Deep vein thrombosis**	453.4, 453.4x, 453.5, 453.5x, 453.72, 453.73, 453.74, 453.75 453.82, 453.83, 453.84, 453.85	I80.1x, I80.2x, I80.3, I80.8, I80.9 I82.4xx, I82.5xx, I82.60x, I82.62x, I82.70x, I82.72x, I82.Axx, I82.Bxx,	
**Pulmonary embolism**	415.1, 415.1x, 416.2	I26, I27.82	
**Intracranial hemorrhage**	430, 431, 432, 852, 853	I60, I61, I62	Discharge
**Gastrointestinal bleeding**	456.0, 456.2, 455.2, 455.5, 455.8, 530.7, 530.82, 531.0–531.6, 532.0–532.6, 533.0–533.6, 534.0–534.6, 535.0–535.6 537.83, 562.02, 562.03, 562.12 562.13 568.81, 569.3, 569.85, 578.0, 578.1, 578.9	K250, K260, K270, K280, K290	Discharge
**Other critical site bleeding**	423,0, 459.0, 568.81, 593.81, 599.7, 623.8, 626.3, 626.6, 719.1, 784.7, 784.8, 786.3	D62, J942, H113, H356, H431, N02, N95, R04, R31, R58	Discharge

© 2021 Chen DY et al. *JAMA Network Open.*

eFigure 1. Enrollment and Follow-Up of Study Patients

Patients with basal cell carcinoma or squamous-cell skin cancer were not included in the study. Index date was defined as the first date of receiving NOACs or enoxaparin. VTE, venous thromboembolism; NOACs, non-vitamin K antagonist oral anticoagulants, LMWH, low molecular weight heparin

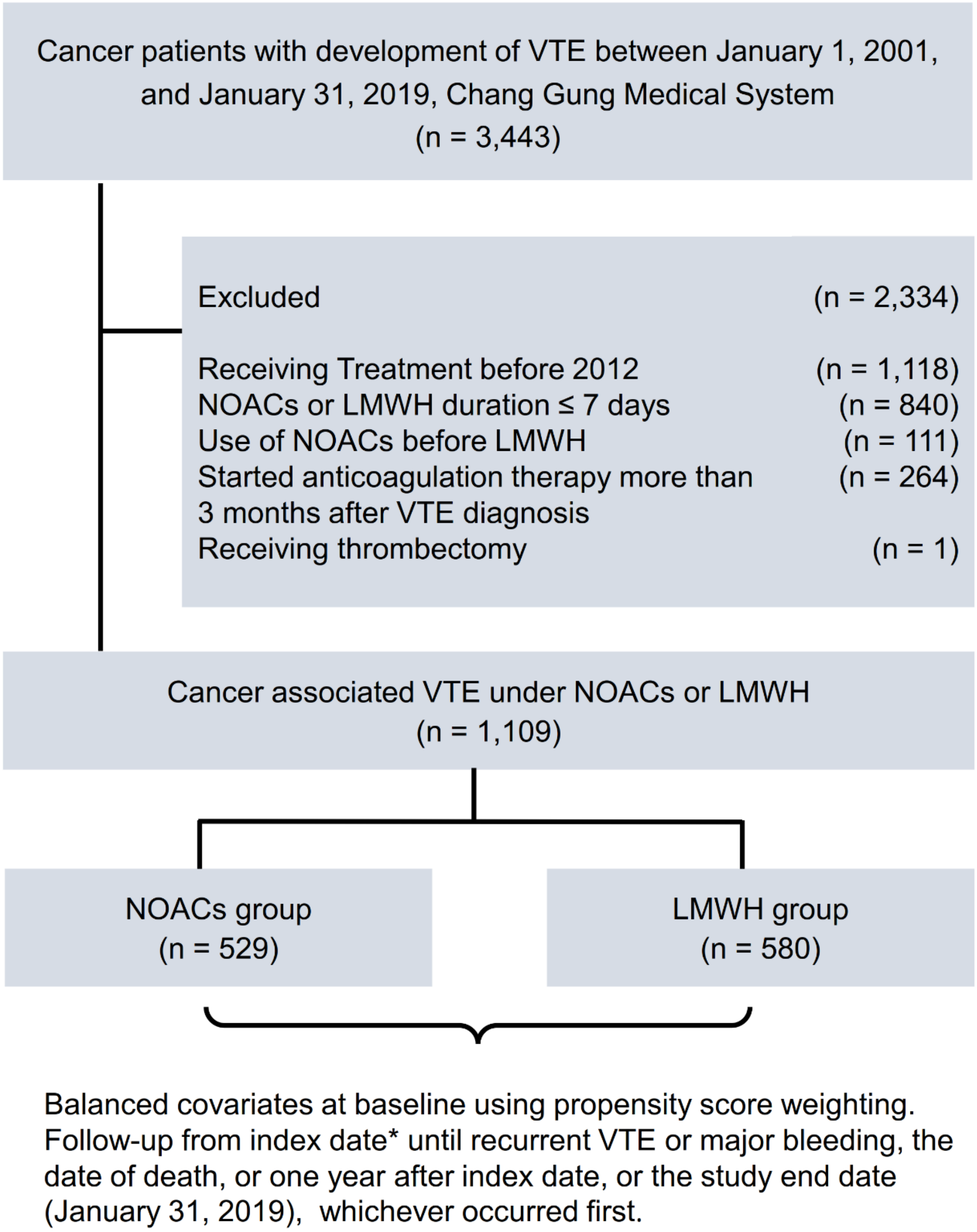

© 2021 Chen DY et al. *JAMA Network Open.*

eFigure 2. Analysis for Subgroups for Outcomes of Recurrent VTE for Patients Treated With NOAC or LMWH

NOACs, non-vitamin K antagonist oral anticoagulants, LMWH, low molecular weight heparin. GI, gastrointestinal

Subgroup	Number of event (%) NOACs	LMWH	HR (95% CI)	*P* interaction
**Gender**				.72
Male	19 (3.6)	31 (5.3)	0.69 (0.36-1.35)	
Female	19 (3.6)	29 (5.0)	0.56 (0.29-1.07)	
**Age (years)**				.11
< 65	11 (2.1)	34 (5.9)	0.38 (0.17-0.82)	
≥ 65	27 (5.1)	26 (4.5)	0.88 (0.48-1.61)	
**Platelet count (per μl)**				<.001
>100,000	20 (3.8)	52 (9.0)	0.37 (0.21-0.67)	
50,000–100,000	3 (0.6)	4 (0.7)	1.22 (0.24-6.29)	
<50,000	15 (2.8)	4 (0.7)	3.72 (1.04-13.36)	
**Hemoglobin level (g/dL)**				.10
>10	17 (3.2)	32 (5.5)	0.50 (0.26-0.98)	
8–10	6 (1.1)	22 (3.8)	0.33 (0.12-0.9)	
<8	15 (2.8)	6 (1.0)	2.43 (0.79-7.54)	
**Tumor type**				.94
GI tract cancer	7 (1.3)	19 (3.3)	0.41 (0.14-1.15)	
Non-GI cancer	31 (5.9)	41 (7.1)	0.70 (0.42-1.19)	
**Stage of cancer**				.05
Early/locally advanced	22 (4.2)	22 (3.8)	0.87 (0.44-1.69)	
Metastatic	9 (1.7)	31 (5.3)	0.39 (0.17-0.89)	

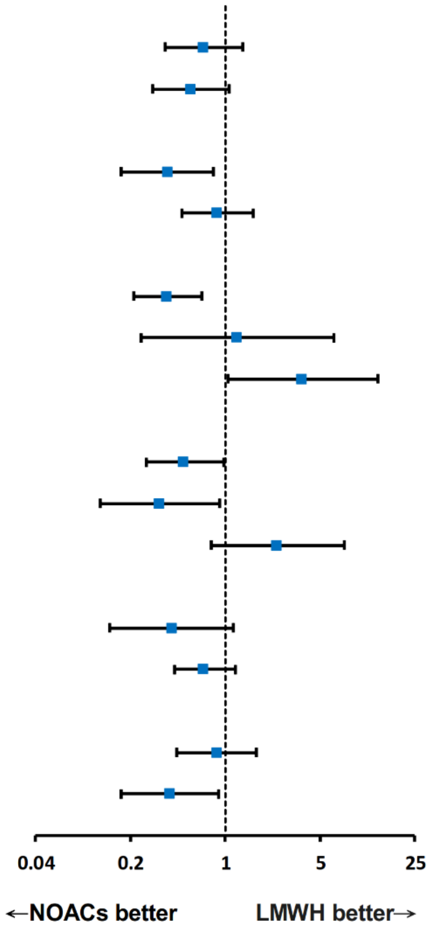

© 2021 Chen DY et al. *JAMA Network Open.*

eFigure 3. Analysis for Subgroups for Outcomes of Major Bleeding for Patients Treated With NOAC or LMWH

NOACs, non-vitamin K antagonist oral anticoagulants, LMWH, low molecular weight heparin. GI, gastrointestinal

Subgroup	Number of event (%) NOACs	Number of event (%) LMWH	HR (95% CI)	*P* interaction
**Gender**				.05
Male	12 (2.3)	27 (4.7)	0.56 (0.27-1.13)	
Female	27 (5.1)	28 (4.8)	1.01 (0.58-1.75)	
**Age (years)**				.93
< 65	17 (3.2)	27 (4.7)	0.80 (0.42-1.51)	
≥ 65	22 (4.2)	28 (4.8)	0.81 (0.45-1.44)	
**Platelet count (per µl)**				.15
>100,000	26 (4.9)	47 (8.1)	0.60 (0.36-0.99)	
50,000–100,000	4 (0.8)	3 (0.5)	3.11 (0.67-14.57)	
<50,000	9 (1.7)	5 (0.9)	1.68 (0.50-5.67)	
**Hemoglobin level (g/dL)**				.55
>10	20 (3.8)	29 (5.0	0.79 (0.44-1.42)	
8–10	7 (1.3)	20 (3.4)	0.47 (0.20-1.13)	
<8	12 (2.3)	6 (1.0)	1.88 (0.65-5.46)	
**Tumor type**				.35
GI tract cancer	5 (0.9)	12 (2.1)	0.48 (0.15-1.52)	
Non-GI cancer	34 (6.4)	43 (7.4)	0.88 (0.55-1.41)	
**Stage of cancer**				.32
Early/locally advanced	17 (3.2)	21 (3.6)	0.70 (0.35-1.39)	
Metastatic	18 (3.4)	29 (5.0)	1.07 (0.58-1.96)	
**Risk factors for bleeding**				.80
0~1	24 (4.5)	27 (4.7)	0.93 (0.54-1.62)	
2	11 (2.1)	23 (4.0)	0.51 (0.22-1.16)	
3	4 (0.8)	5 (0.9)	1.34 (0.32-5.53)	

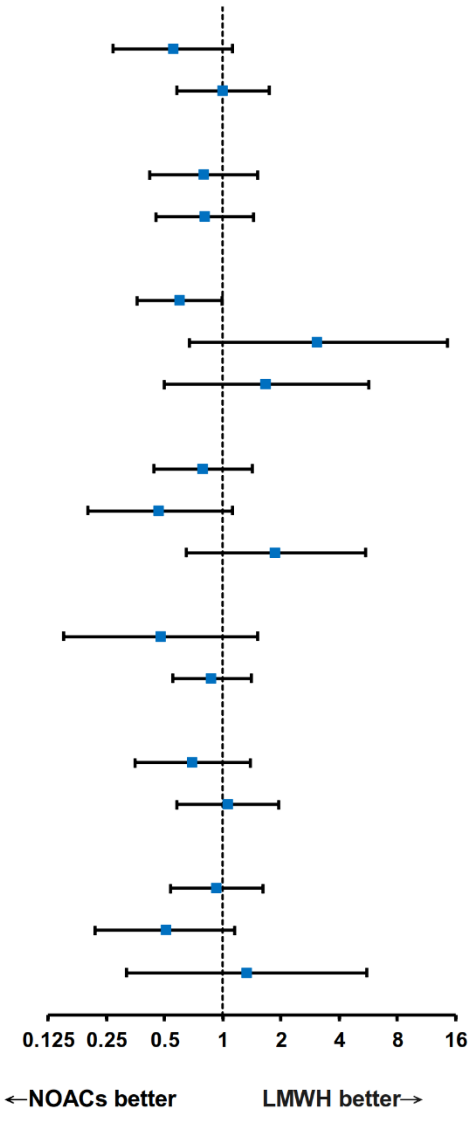

© 2021 Chen DY et al. *JAMA Network Open.*

eFigure 4. Analysis for Subgroups for Outcomes of Major GI Bleeding for Patients Treated With NOACs or LMWH

NOACs, non-vitamin K antagonist oral anticoagulants, LMWH, low molecular weight heparin. GI, gastrointestinal

Subgroup	Number of event (%) NOACs	LMWH	HR (95% CI)	*P* interaction
**Gender**				.84
Male	4 (0.8)	20 (3.4)	0.30 (0.10-0.84)	
Female	6 (1.1)	21 (3.6)	0.29 (0.11-0.74)	
**Age (years)**				.30
< 65	3 (0.6)	20 (3.4)	0.20(0.06-0.68)	
≥ 65	7 (1.3)	21 (3.6)	0.36 (0.16-0.85)	
**Platelet count (per µl)**				.43
>100,000	7 (1.3)	36 (6.2)	0.24 (0.11-0.52)	
50,000–100,000	0 (0)	2 (0.3)	-	
<50,000	3 (0.6)	3 (0.5)	1.15 (0.21-6.46)	
**Hemoglobin level (g/dL)**				.93
>10	6 (1.1)	23 (4)	0.33 (0.13-0.79)	
8–10	1 (0.2)	15 (2.6)	0.09 (0.01-0.61)	
<8	3 (0.6)	3 (0.5)	1.01 (0.18-5.54)	
**Tumor type**				.31
GI tract cancer	3 (0.6)	10 (1.7)	0.41 (0.11-1.57)	
Non-GI cancer	7 (1.3)	31 (5.3)	0.26 (0.12-0.60)	
**Stage of cancer**				.55
Early/locally advanced	5 (0.9)	16 (2.8)	0.27 (0.09-0.81)	
Metastatic	5 (0.9)	20 (3.4)	0.48 (0.19-1.23)	
**Risk factors for bleeding**				.18
0~1	5 (0.9)	21 (3.6)	0.26 (0.10-0.67)	
2	3 (0.6)	17 (2.9)	0.20(0.05-0.79)	
3	2 (0.4)	3 (0.5)	1.31 (0.20-8.74)	

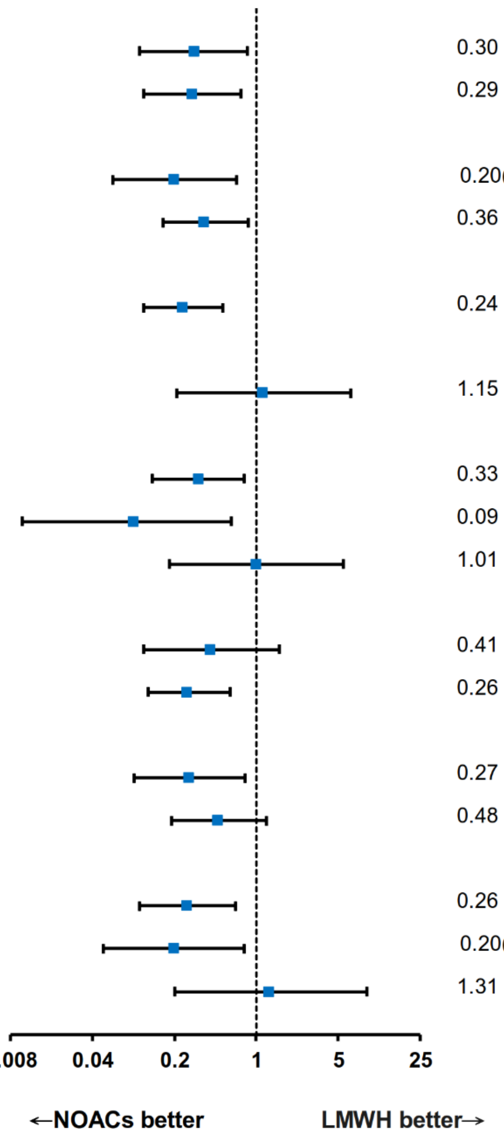

© 2021 Chen DY et al. *JAMA Network Open.*

eFigure 5. Stratified Analysis by Anticoagulant Duration

m, months

Subgroup	Number of event (%) NOACs	LMWH	HR (95% CI)	*P* interaction
**Recurrent VTE**				.07
<3m	12 (2.3)	41 (7.1)	0.50 (0.24-1.03)	
≥3m	26 (4.9)	19 (3.3)	0.44 (0.22-0.86)	
**Major bleeding**				.71
<3m	19 (3.6)	40 (6.9)	0.92 (0.53-1.62)	
≥3m	20 (3.8)	15 (2.6)	0.57 (0.28-1.19)	
**Recurrent VTE or major bleeding**				.57
<3m	31 (5.9)	70 (12.1)	0.84 (0.54-1.31)	
≥3m	44 (8.3)	31 (5.3)	0.49 (0.29-0.81)	

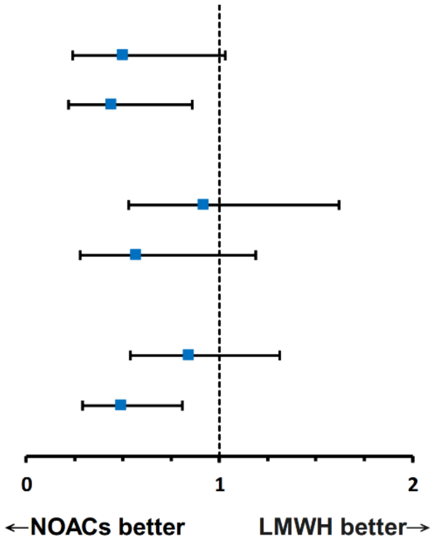

© 2021 Chen DY et al. *JAMA Network Open.*
